# Curcumin Targeting NF-*κ*B/Ubiquitin-Proteasome-System Axis Ameliorates Muscle Atrophy in Triple-Negative Breast Cancer Cachexia Mice

**DOI:** 10.1155/2022/2567150

**Published:** 2022-01-29

**Authors:** Jin Zhang, Jin Zheng, Haitao Chen, Xinrong Li, Chenxiao Ye, Fan Zhang, Zewei Zhang, Qinghua Yao, Yong Guo

**Affiliations:** ^1^The First Clinical Medical College, Zhejiang Chinese Medical University, Hangzhou 310053, China; ^2^Department of Oncology, Hospital Affiliated to Shaanxi University of Chinese Medicine, Xianyang 712000, China; ^3^Department of Traditional Chinese Medicine, The Second Hospital Affiliated to Air Force Medical University, Xi'an 710038, China; ^4^Department of Integrated Traditional Chinese and Western Medicine, Cancer Hospital of the University of Chinese Academy of Sciences/Zhejiang Cancer Hospital, Hangzhou 310022, China; ^5^Department of Hepatopancreatobiliary Surgery, Cancer Hospital of the University of Chinese Academy of Sciences/Zhejiang Cancer Hospital, Hangzhou 310022, China; ^6^Key Laboratory of Traditional Chinese Medicine Oncology, Zhejiang Cancer Hospital, Hangzhou 310022, China; ^7^Department of Oncology, The First Affiliated Hospital of Zhejiang Chinese Medical University, Hangzhou 310003, China

## Abstract

**Background:**

Curcumin is a polyphenol plant-derived compound with anti-inflammatory, antioxidant stress, and anticancer properties that make it have the potential to treat cancer cachexia. However, the role of it in breast cancer cachexia remains unclear.

**Methods:**

The 4T1 cells were subcutaneously injected into BALB/c mice to induce breast cancer cachexia. After tumor formation, the animals were divided into groups and given curcumin or saline interventions. The therapeutic effect of curcumin on breast cancer cachexia was characterized by tumor growth, changes in body mass and gastrocnemius mass, muscle function test, histopathology, and serum nutrition indexes. Mitochondrial function in muscle tissue was observed by transmission electron microscopy and ATP detection, muscle inflammatory factors were detected by ELISA, muscle differential metabolites were detected by ^1^HNMR metabolomics, and the muscle tissue ubiquitination levels and NF-KB expression were also analyzed by RT-qPCR and Western blot.

**Results:**

Dynamic *in vivo* bioluminescence imaging find that curcumin inhibited the growth of tumor in triple-negative breast cancer- (TNBC-) bearing mice, slowed down the loss of body weight and gastrocnemius weight, corrected the mitochondrial dysfunction and malnutrition status, and also significantly improved skeletal muscle function. ELISA analysis found that the level of inflammatory factors in muscle tissue was reduced. ^1^HNMR metabolomics analysis suggested that curcumin could regulate energy metabolism pathways. RT-qPCR and Western blot analysis found that the expression of myogenic factor myogenin was increased and the expression of myodegradation factor myostatin was decreased in the gastrocnemius; the level of ubiquitination and activation of the NF-*κ*B pathway were also declined.

**Conclusions:**

Curcumin reduces ubiquitination, inflammation in skeletal muscle by regulating the NF-KB/UPS axis and improves muscle malignant metabolic phenotype and mitochondrial dysfunction, to alleviate muscle atrophy and loss of function in mice with breast cancer cachexia.

## 1. Introduction

Breast cancer is the most common female tumor disease; its morbidity and mortality rates are increasing year by year, resulting in a serious social and economic burden. Triple-negative breast cancer (TNBC) is a common but aggressive type of breast cancer with limited treatment options [1, 2]. TNBC is characterized by high malignancy, strong aggressiveness, and poor prognosis, and it has more chance to develop cancer cachexia. According to statistics, cachexia is present in more than half of cancer patients, contributing about 40% of cancer-related deaths [3]. The pathogenesis of cancer cachexia involves metabolic disorder, chronic inflammation, and skeletal muscle protein degradation [4, 5]. Weight loss and skeletal muscle atrophy not only are common features of cachexia in cancer patients but also are independent prognostic factors for poor clinical outcomes [6]. Unfortunately, many cancer patients often lack effective treatments [7, 8]; current interventions such as exercise, nutritional supplements, and drugs have not achieved the desired results in reducing muscle wasting [9]. Therefore, it is imperative to explore the effective methods and drugs to prevent cancer cachexia [10].

Skeletal muscle wasting caused by tumor cachexia is a gradual loss of muscle mass and strength [11], mostly due to decreased protein synthesis and/or increased degradation [6, 12]. As a protein degradation pathway, the ubiquitin-proteasome system (UPS) plays an important role in the occurrence of skeletal muscle atrophy [13]. Ubiquitin binds to the substrate of the target protein through ubiquitin-activating enzymes E1, E2, and E3 and activates the proteolytic signaling pathway, for example, the E3 ubiquitin ligase atrogin-1/MAFbx and MuRF1 genes are increased in atrophic skeletal muscle [14–16]. UPS can also mediate mitochondrial dysfunction and lead to skeletal muscle atrophy in cancer patients [17]. Studies have reported that the activation of NF-*κ*B can increase the expression of atrogin-1/MAFbx and MuRF1 in muscle atrophy models; NF-*κ*B also inhibits the expression of mitochondrial genes, thereby reducing mitochondrial biosynthesis [18, 19]. Tumor inflammatory cytokines such as TNF-*α* and IL-6 can also increase protein degradation by activating NF-*κ*B and UPS pathways to promote skeletal muscle atrophy [20, 21]. More importantly, muscle consumption is critically correlated with the muscle metabolism disorder; the expression levels of glucose, amino acids, and fatty acids in muscle can be changed with chemotherapy [22, 23].

Curcumin is a diketone compound extracted from the dried roots of *Curcuma longa* l. and widely used as a traditional herb medicine in Asia [24, 25]. It has profound anti-inflammatory and anticancer properties and can be served as a NF-*κ*B inhibitor to inhibit inflammatory cytokines and oxidative stress in various diseases [25]. However, limited scientific evidence on the mechanism and efficacy of curcumin hinders its integration into the mainstream of healthcare. As the public's interest on natural phytotherapy and diet therapy related to health and disease increases, this attractive and safe alternative has been extensively studied. Previous studies have also shown that curcumin alleviates postexercise skeletal muscle injury by inhibiting the activity of the ubiquitin-proteasome system [26]. Therefore, we hypothesize that curcumin may be able to improve breast cancer cachexia-related skeletal muscle atrophy.

**Figure 1:**
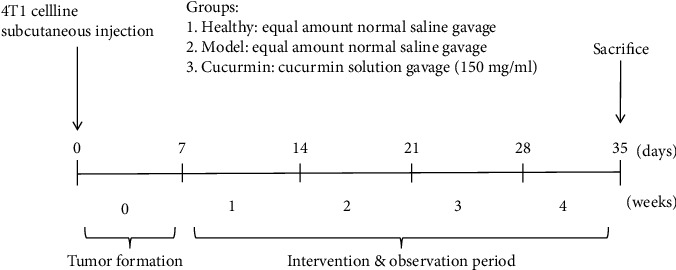
Schematic diagram of the research process.

Hence, in this study, by establishing a TNBC-cachexia mouse model, we performed a series of experiments to investigate the effect of curcumin on cancer cachexia-related skeletal muscle atrophy. We find that curcumin inhibited the growth of tumors and corrects mitochondrial dysfunction and malnutrition status in muscle fibers, as well as skeletal muscle function significant improvement. Further results indicated that curcumin improved the muscle atrophy in TNBC-cachexia mice by regulating the NF-*κ*B/UPS axis. Our study is expected to provide to novel perspective for the nature compound as a candidate agent for cancer cachexia therapy. The schematic diagram of the research is shown in Figure 1.

## 2. Materials and Methods

### 2.1. Animal Experiment

Female, 5-week-old BALB/c mice (Zhejiang Chinese Medical University Laboratory Animal Research Center, Hangzhou, China) were given autoclaved chow and water ad libitum under a 12 h light/dark cycle at a room temperature of 24 ± 2°C and humidity of 55 ± 10%. After 7 days of acclimatization, the mice were randomly divided into three groups (*n* = 8/group): healthy group, model group, and curcumin group. Mice in the model group and the curcumin group were inoculated with 5 × 10^5^ 4T1 cells (Cell Bank of the Chinese Academy of Sciences, Shanghai, China); the subcutaneous tumor was formed for 1 week, and treatment intervention began. Mice in the curcumin group were intragastrically treated with 0.2 mL curcumin solution (Sigma, Saint Louis, USA) of 150 mg/mL for 28 days, while the healthy and model groups received an equal amount of normal saline. Record weight and food intake as planned. After 28 days of treatment intervention, experimental samples were collected for the experiment. All animal experiments were conducted with the approval of the Experimental Animal Ethical Committee of the Zhejiang Chinese Medical University.

### 2.2. Activity Monitoring

On day 28 of intervention, all mice were tested one by one in a 100 × 100 cm^2^ open field facility in random order. Before testing, the arena was cleaned and dried with 75% alcohol. All spontaneous activities were recorded by a motion tracking system (Sony Co., Ltd. Guangzhou, China) for 5 minutes. The experimental results were analyzed using SMART 3.0 (Panlab, Barcelona, Spain), and the total movement distance was used as the evaluation index of athletic ability.

### 2.3. Muscle Grip Strength Test

The grasping strength of the forelimbs of the mice was measured with a grip strength tester (YLS-13A, Shandong Academy of Medical Sciences, China) on days 0, 14, and 28 after treatment intervention. To reduce variability associated with the operation, the same mice were repeated 5 times and the top 3 peak force measurements were included in the analysis (Unit: gf (gram force)).

### 2.4. Histopathological Assessment

The gastrocnemius muscle was fixed with 4% formalin and further embedded in paraffin and then cut into 4 *μ*m thick sections along the transverse direction of muscle filaments. The gastrocnemius muscle sections were stained with hematoxylin-eosin (HE), and the images were observed and captured under a microscope.

### 2.5. Transmission Electron Microscopic (TEM) Observation

Prepare gastrocnemius blocks and immediately soak them with 2.5% glutaraldehyde. After 6-8 hours at 4°C, cut into 1 mm thick coronal slices. The samples were washed with PBS and fixed with osmium tetroxide for 1-2 hours. After gradient dehydration with alcohol and acetone, the gastrocnemius muscle was embedded in epoxy resin and sliced into ultrathin sections. Images were obtained by transmission electron microscopy (HT7700, Hitachi, Tokyo, Japan) after double staining with uranyl acetate and lead citrate.

### 2.6. Gastrocnemius ATP Content Detection

The ATP content in the gastrocnemius muscle of experimental mice was detected using the ATP detection kit (Beyotime, Shanghai, China) for luminescence detection according to the manufacturer's instructions. All luminescence values are finally standardized by the corresponding protein concentration.

### 2.7. Biochemical Test

Total protein, cholesterol, triglycerides, and albumin were tested by Zhejiang Chinese Medical University Laboratory Animal Research Center using an automatic biochemical analyzer (TBA-40, TOSHIBA, Japan).

### 2.8. Enzyme-Linked Immunosorbent Assay (ELISA)

Weigh the gastrocnemius tissue sample, homogenize it with a lysis buffer containing protease inhibitors on ice, and then centrifuge at 5000 *g* for 5 minutes at 4°C. Collect the supernatant and store it at -20°C. The concentration of IL-6 and TNF-*α* was determined according to the manufacturer's protocol using IL-6 and TNF-*α* ELISA kit (Elabscience, Wuhan, China).

### 2.9. Gastrocnemius Tissue Sample Preparation for Metabolic Profiling

Take out the gastrocnemius muscle from the liquid nitrogen, accurately weigh 100 mg, and add 800 *μ*L MeOH/H_2_O solution (*v*/*v*, 3 : 1). Then, use an electric homogenizer to homogenize at a frequency of 1/30 for 5 minutes to extract polar metabolites. Centrifuge at 4°C and 13,000 rpm for 10 min, and collect the supernatant. Then, 500 *μ*L of supernatant was dried in a vacuum centrifugal concentrator (Labconco, USA) and resuspended in 600 *μ*L of 10% deuterium water (D2O, 99.8%, Sigma, USA) and 0.05 mM sodium 3-trimethylsilyl-propionate-d4 (TMSP-2,2,3-d4; Sigma, USA) for nuclear magnetic resonance analysis.

### 2.10. ^1^H NMR Spectroscopy Analysis and Data Processing

600 *μ*L supernatants from each sample were introduced for NMR spectroscopic analysis performed on a Bruker 600 MHz AVANCE III spectrometer equipped with a 5 mm BBFO probe and lock performed on the D2O signal at 25°C. ^1^H NMR spectra were received using NOESYPR1D pulse sequence with water suppression. The data were processed using Bruker Topspin 3.2. All free induction decays (FIDs) from ^1^H NMR of the muscle were multiplied by a 0.3 Hz exponential line broadening before Fourier transformation. All the obtained NMR spectra were manually phased, baseline corrected, and chemical shift referenced to TSP (*δ* = 0.0) within MestReNova 6.1 (Mestrelab Research SL, Spain). The analyzed spectrum region was 0.0–9.0 ppm without 4.5–5.0 ppm to eliminate the effects of imperfect water suppression. The characteristic peaks of all muscle metabolites were determined based on the network database of metabolomics, including Biological Magnetic Resonance Bank (https://www.bmrb.wisc.edu/metabolomics) and Human Metabolome Database (https://www.hmdb.ca/). The pathway enrichment analysis of differential metabolites is performed in the MetaboAnalyst 5.0 database (https://www.metaboanalyst.ca/).

### 2.11. Real-Time qPCR

RNA was extracted from the muscle samples using RNA-Quick Purification Kit (esunbio, Shanghai, China) according to the manufacturer's protocol, and the RNA was reverse transcribed into cDNA by RevertAid First Strand cDNA Synthesis Kit (Thermo Scientific™, Massachusetts, USA). Real-time PCR (RT-PCR) analyses were performed by the ABI 7900HT real-time PCR system (Applied Biosystems, San Francisco, USA); use Super SYBR Green qPCR Master Mix (esunbio, Shanghai, China). The *β*-actin expression was used to normalize the expression levels, and results were calculated based on the comparative cycle threshold method (2^-*ΔΔ*ct^). The primer sequences are listed in Table 1 (5′−3′).

### 2.12. Western Blotting

About 80 mg gastrocnemius tissue was lysed in RIPA Lysis Buffer (Cell Signaling, Boston, USA) for 10 minutes on ice. The lysate homogenate was centrifuged, and the protein concentration was measured with the BCA Protein Assay Kit (Beyotime, Shanghai, China). After that, the protein samples were loaded and electrophoresed in 10% SDS-PAGE and transferred to PVDF membranes (Millipore, United States). After that, the membranes were blocked in 5% nonfat milk powder for 2 h before incubation with primary antibodies overnight at 4°C. The primary antibody was purchased from Abcam (Cambridge, UK) and diluted at 1 : 1000, including anti-GAPDH, anti-GDF8/myostatin, anti-MuRF1, anti-atrogin-1 (Fbx32), anti-NF-*κ*B (p65), and anti-NF-*κ*B p65 (phosphorylation S536). Then, the membranes were incubated with the appropriate secondary antibody. The blots were visualized with enhanced chemiluminescence.

### 2.13. Statistical Analysis

The partial least-squares discriminant analysis (PLS-DA) was performed using SIMCA software, version 14.1 The differential metabolites were filtered by variable influence on projection (VIP) selection according to the PLS-DA and the filtering conditions VIP > 1 and *p* < 0.05. Differences between the two groups were analyzed by unpaired Student's *t*-test. All values were expressed as mean ± SEM. *p* < 0.05 was considered statistically significant.

## 3. Result

### 3.1. Curcumin Inhibited the Tumor Growth of TNBC-Bearing Mice

**Figure 2:**
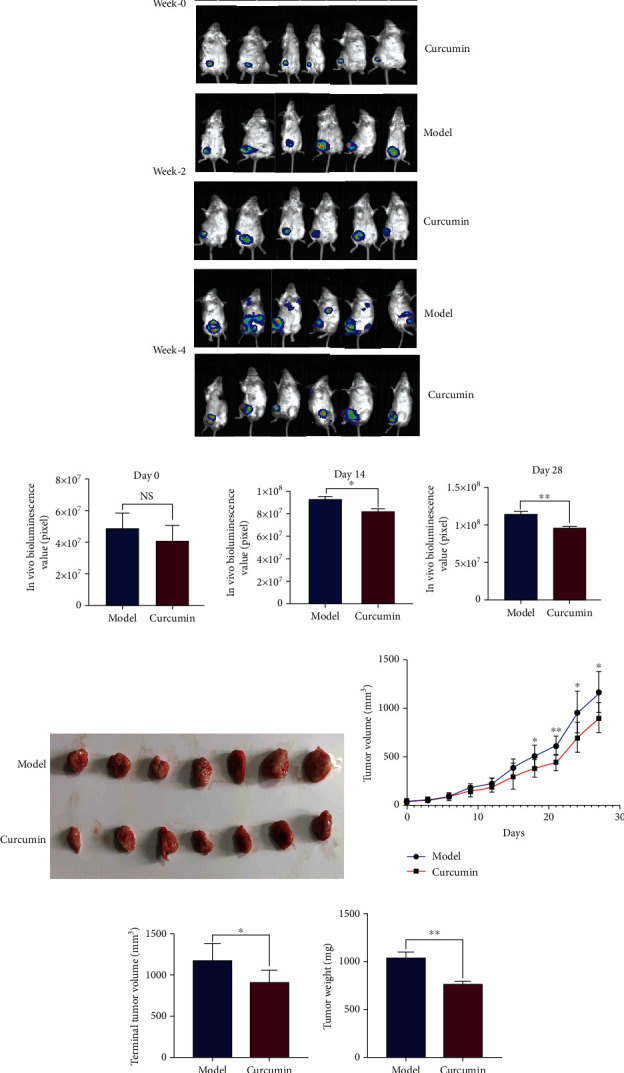
Curcumin inhibits the proliferation of triple-negative breast cancer. (a) In vivo bioluminescence imaging of the breast cancer-bearing mice on day 0, day 14, and day 28 after intervention (*n* = 6/group). (b) Statistical analysis of bioluminescence value of tumor-bearing mice on day 0, day 14, and day 28 after intervention (*n* = 6/group). (c) Comparison of in situ tumor morphology between tumor-bearing model mice and curcumin treatment mice. (d) Model group and curcumin treatment group tumor growth curve during the intervention period (*n* = 8/group). (e, f) Statistical analysis of terminal tumor volume and bodyweight in the model group and the curcumin group (*n* = 8/group). NS: not significant; ^∗^*p* < 0.05 and ^∗∗^*p* < 0.01 by *t*-test, compared to the model group. Data were shown as mean ± standard errors of the mean.

We used dynamic *in vivo* bioluminescence imaging monitoring to evaluate the mouse growth changes of breast cancer tumor at the beginning, middle, and end of the experiment (Figure 2(a)). *In vivo* bioluminescence imaging results showed that the bioluminescence values of tumor-bearing mice in the curcumin group were significantly lower than those of the model control group at 2 and 4 weeks (Figure 2(b), *p* = 0.0205 < 0.05; *p* = 0.0056 < 0.01). In addition, as shown in Figures 2(c)–2(f), it could also be found that the tumor volume of the curcumin group mice was significantly smaller than that of the model group, and the tumor weight of the mice also reached the same conclusion. These data strongly suggest that curcumin inhibits the tumor growth of breast cancer.

### 3.2. Curcumin Alleviated Body Weight Loss and Improved Malnutrition in TNBC-Bearing Mice

**Figure 3:**
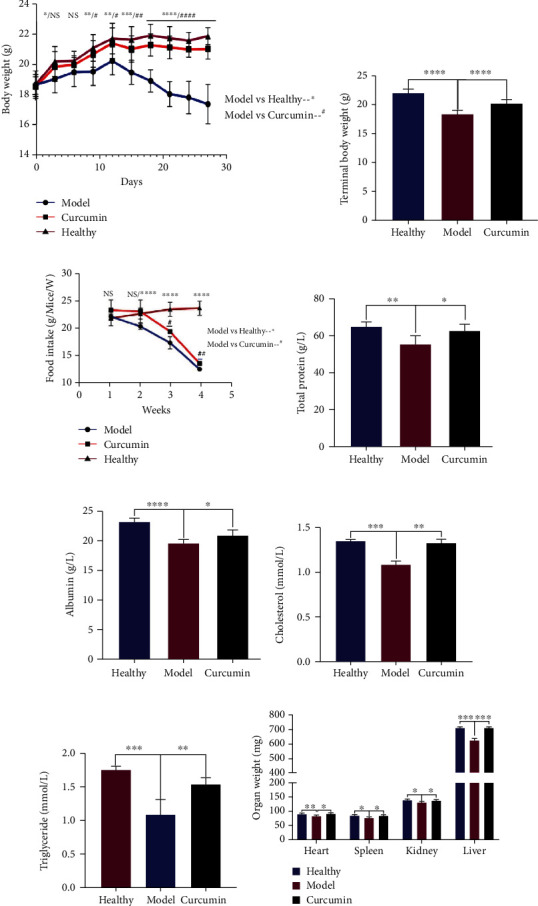
Curcumin alleviates the weight and organ loss and improved malnutrition in tumor-bearing mice. (a) Body weight change curves of the three groups during the 4-week intervention period. (b) Statistical analysis of the terminal body weight of three groups of mice. (c) Statistical analysis of the food intake of the 3 groups of mice during the 4-week observation period. (d–g) Statistical analysis of serum total protein, albumin, cholesterol, and triglyceride of mice in the healthy group, the model group, and the curcumin group at the end of the experiment. (h) Statistical analysis of the weight of the main organs of the three groups of mice at the end of the experiment. *n* = 8/group. NS: not significant; ^∗^*p* < 0.05, ^∗∗^*p* < 0.01, ^∗∗∗^*p* < 0.001, and ^∗∗∗∗^*p* < 0.0001 by *t*-test, compared to the model group. Data was shown as mean ± standard errors of the mean.

From the ninth day until the end of the experiment, the bodyweight of the tumor-bearing mice in the curcumin group was significantly larger than that of the model group, but lower than that of the healthy group, as well as the final bodyweight (Figures 3(a) and 3(b)). In addition, the weekly eating status and some nutritional indicators of each group mice were also evaluated. The results showed that the average weekly food intake of mice in the healthy group and the curcumin group was greater than that of the model group within 4 weeks, with statistical difference in the 3rd and 4th weeks (Figure 3(c), *p* < 0.05). The level of nutritional indicators of mice in the model group was significantly lower than that in the healthy group, including total protein, albumin, cholesterol, and triglyceride (Figures 3(d)–3(g), *p* < 0.01), while these indicators were significantly improved after curcumin treatment, including total protein (Figure 3(d), *p* = 0.0249), albumin (Figure 3(e), *p* = 0.0131), cholesterol (Figure 3(f), *p* = 0.0011), and triglyceride (Figure 3(g), *p* = 0.0039). In addition, the weight of the main organs of mice in the model group was significantly lower than that of the healthy group, while curcumin administration partly recovered these organs' weights (Figure 3(h), *p* < 0.05).

### 3.3. Curcumin Reduced the Skeletal Muscle Loss in Tumor-Bearing Mice and Improves Muscle Function

**Figure 4:**
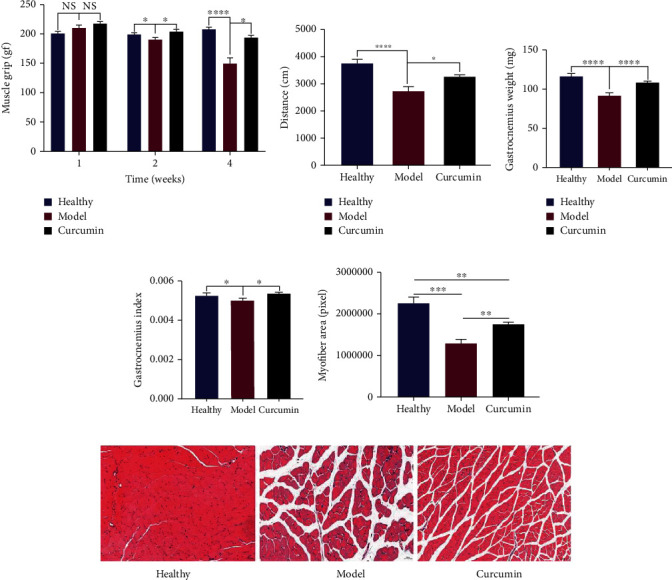
Curcumin reduces skeletal muscle loss and improved muscle function in tumor-bearing mice. (a) Statistical analysis of forelimb grip strength of three groups of mice at 0, 2, and 4 weeks after intervention (*n* = 8/group). (b) Statistical analysis of movement distance within 5 min in three groups of mice at the end of the experiment (*n* = 8/group). (c) Statistical analysis of gastrocnemius muscle weight in three groups of mice at end of the experiment (*n* = 8/group). (d) Comparative analysis of the gastrocnemius index of the three groups of mice at the end of the experiment (*n* = 8/group). (e) Statistical analysis of muscle fiber cross-sectional area in gastrocnemius HE sections of three groups of mice (*n* = 5/group). (f) HE staining analysis of gastrocnemius muscle in three groups (×200 magnification; scale bar: 50 *μ*m, *n* = 5/group). NS: not significant; ^∗^*p* < 0.05, ^∗∗^*p* < 0.01, ^∗∗∗^*p* < 0.001, and ^∗∗∗∗^*p* < 0.0001 by *t*-test, compared to the model group. Data was shown as mean ± standard errors of the mean.

We measured the grip strength of the forelimbs of the three groups of mice. Compared with that of the healthy group, the grip strength of the model group mice was decreased rapidly, while curcumin administration enhanced the gripping strength, with statistical differences at 2 and 4 weeks (Figure 4(a), curcumin vs. model: *p* = 0.047 and *p* = 0.0011, respectively). In addition, Figure 4(b) indicates that the movement distance of the model group mice was significantly shorter than that of the healthy group (*p* < 0.0001), while this in the curcumin group was significantly decreased (Figure 4(b), *p* = 0.0102). Furthermore, we found that the reduced gastrocnemius weight in the model group mice was significantly recovered by curcumin administration (Figure 4(c), *p* < 0.0001). The lowered gastrocnemius index of the model group mice was also recovered in the curcumin group (Figure 4(d), *p* = 0.0214).

In Figures 4(e) and 4(f), HE staining analysis showed that the healthy group muscle fibers were evenly arranged, showing a dense polygonal structure with regular muscle spaces. The muscle fibers in the model group were different in thickness, with an increased diameter and disordered arrangement, and the gaps were increased significantly after muscle fiber atrophy. The cross-sectional area of muscle fibers was also significantly smaller than that in the healthy group (Figure 4(e), *p* < 0.0001). Compared with the model group, the muscle fiber density and area of the mice in the curcumin group were significantly increased, the arrangement was orderly, the muscle fiber gap was significantly reduced (Figure 4(e), *p* = 0.0055), and the condition of muscle atrophy was improved.

### 3.4. Curcumin Alleviates Mitochondrial Dysfunction and Increases ATP Production in the Gastrocnemius Tissue of Tumor-Bearing Mice

**Figure 5:**
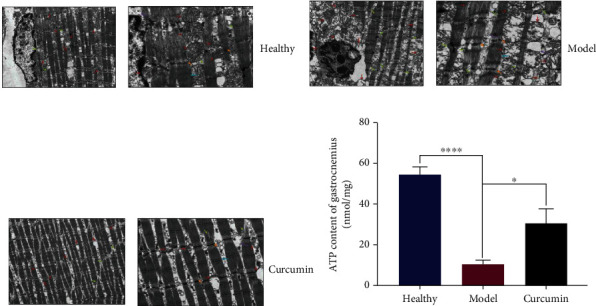
Curcumin alleviates mitochondrial dysfunction and increased ATP production in gastrocnemius of TNBC mice. (a–c) Transmission electron microscope observation of the gastrocnemius in the healthy group, the model group and the curcumin group (green: myofibrils; orange: sarcomere Z-line; blue: H band; reddish-brown: mitochondria; purple: edema; left: 20,000x magnification; right: 50,000x magnification). (d) Statistical analysis of ATP content in gastrocnemius tissue of mice in three groups (*n* = 4/group). ^∗^*p* < 0.05 and ^∗∗∗∗^*p* < 0.0001 by *t*-test, compared to the model group. Data were shown as mean ± standard errors of the mean.

TEM was used to examine the ultrastructure of muscle fibers to assess mitochondrial damage. The result in Figure 5(a) showed that the nuclei (reddish-brown) in the muscle fibers of the mice of the healthy group did not exhibit pyknosis, the mitochondria did not aggregate, and they were evenly distributed between myofibrils (green), mitochondria (reddish-brown) have regular morphology, myofibrils (green) line up neatly, sarcomeres Z-line (orange), H-band (blue) clear. In the model group, Figure 5(b) shows nuclei pyknosis separated from the cytoplasm, mitochondrial aggregation, injury swelling and expansion (reddish-brown), myofibrils (green) broken, sarcomere blurred, sarcomere Z-line (orange), sarcomere H-band (blue) blurred, edema, and voids (purple). In the curcumin group (Figure 5(c)), the number of mitochondria (reddish-brown) was reduced compared with that in the healthy group, and the swelling was obvious. The mitochondrial ridges disappeared and vacuoles were formed, distributed among the myofibrils, but no mitochondrial aggregation was observed. The myofibrils were in normal shape, and the overall shape was slightly worse than that in the normal group. The myofibrils (green) are arranged in a continuous and orderly manner, with no apparent rupture. In addition, the ATP content in the gastrocnemius of the model group is significantly lower than that in the control group, and curcumin treatment improved ATP content (Figure 5(d), *p* < 0.05).

### 3.5. Curcumin Intervention Alleviated the Malignant Metabolic Phenotype of Muscle Tissue in Tumor-Bearing Mice

**Figure 6:**
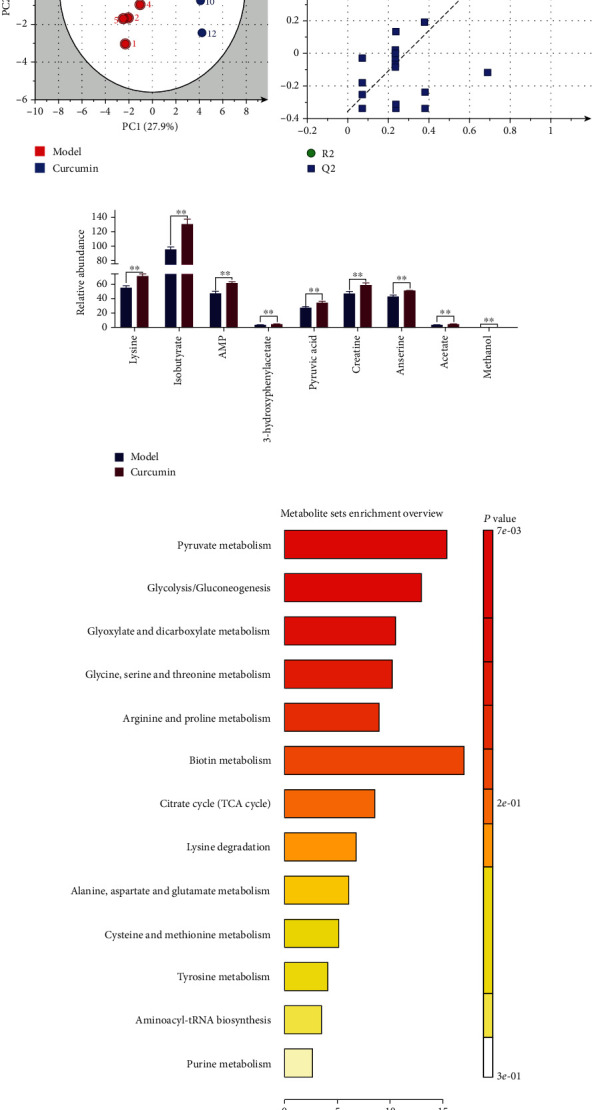
Metabolomics analysis of gastrocnemius tissue in tumor-bearing mice after curcumin intervention. (a) PLS-DA scatter plot of gastrocnemius samples in the model group and the curcumin group (*n* = 8/group). (b) Validation plot of gastrocnemius samples obtained from the model and curcumin groups (*n* = 8/group). (c) Gastrocnemius differential metabolites filtered by the conditions of VIP > 1 and *p* < 0.05. (d) Pathway analysis of differential metabolites; ^∗∗^*p* < 0.01 by *t*-test, compared to the model group. Data were shown as mean ± standard errors of the mean.

The above results indicate that curcumin significantly improved the cachexia phenotype of the skeletal muscle of TNBC tumor-bearing mice. To further analyze the underlying mechanism, we performed ^1^HNMR metabonomic analysis on the gastrocnemius tissue, and the unsupervised partial least-squares discriminant analysis (PLS-DA) score plots showed that the curcumin group and the model group have a good degree of discrimination, which also shows that the metabolic patterns between the two groups are different (Figure 6(a)). The verification plots based on the two sets of ^1^HNMR spectra illustrated that the PLS-DA model is robust and reliable (Figure 6(b), the R2X, R2Y, and Q2 values of the model are 0.551, 0.989, and 0.889, respectively). Then, six different metabolites were screened according to the conditions of VIP > 1 and *p* < 0.05, including lysine, isobutyrate, AMP, 3-hydroxyphenylacetate, pyruvic acid, and creatine (Figure 6(c)). Based on the differential metabolites, related metabolic pathways were identified by KEGG and HMDB databases (Figure 6(d)). We specifically analyzed the top three metabolic pathways with significant differences, including pyruvate metabolism, glycolysis/gluconeogenesis, and glyoxylate and dicarboxylate metabolism, and these pathways are considered to be the metabolic pathways most relevant to curcumin treatment (for more information, please see supplementary data (Table S1)).

### 3.6. Curcumin Decreased the Inflammatory Factors and Myodegradable Factors and Increased the Myogenic Factor Expression in the Muscle Tissues

**Figure 7:**
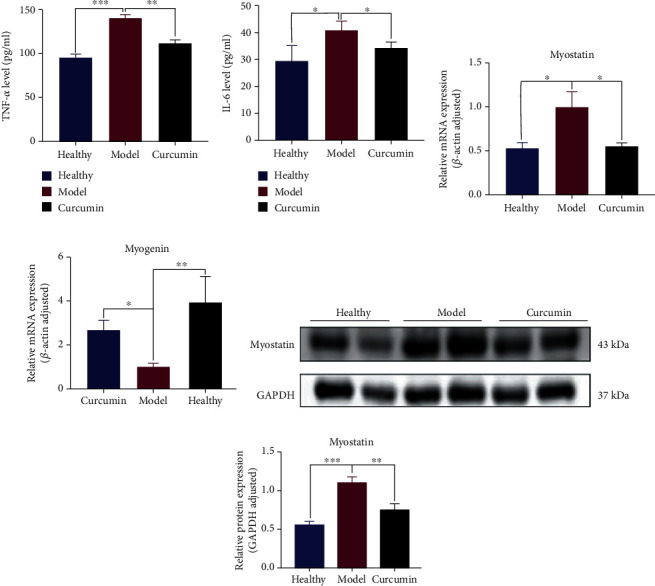
Curcumin decreased inflammatory factors, increased the expression of myogenic factors, and decreased the expression of myodegradable factors in the muscle tissue of tumor-bearing mice. (a, b) Statistical analysis of TNF-*α* and IL-6 levels in gastrocnemius of three groups of mice (*n* = 4/group). (c, d) Statistical analysis of myostatin and myogenin mRNA expression in gastrocnemius of mice in three groups. (e, f) Statistical analysis of myostatin and myogenin protein expression difference in gastrocnemius of the three groups. NS: not significant; ^∗^*p* < 0.05, ^∗∗^*p* < 0.01, and ^∗∗∗^*p* < 0.001 by *t*-test, compared to the model group. Data was shown as mean ± standard errors of the mean.

We measured the levels of inflammatory cytokines TNF-*α* and IL-6 in gastrocnemius tissue; the results indicated that the increased levels of TNF-*α* and IL-6 levels in the model group were significantly decreased by curcumin administration (Figures 7(a) and 7(b); *p* = 0.0006, *p* = 0.016). RT-qPCR results showed that the expression of the negative regulator myostatin of muscle in the model group was significantly higher than that of the healthy group (Figure 7(c), *p* = 0.0397), while the myostatin of the curcumin group was less than that in the model group (Figure 7(c), *p* = 0.0496), while the expression of myogenin had opposite results in these groups (Figure 7(d)). The results of myostatin protein expression in Figure 7(e) were consistent with Figure 7(c).

### 3.7. Curcumin Treatment Reduced Ubiquitination and NF-*κ*B Activity in Muscle Tissue of Tumor-Bearing Mice

**Figure 8:**
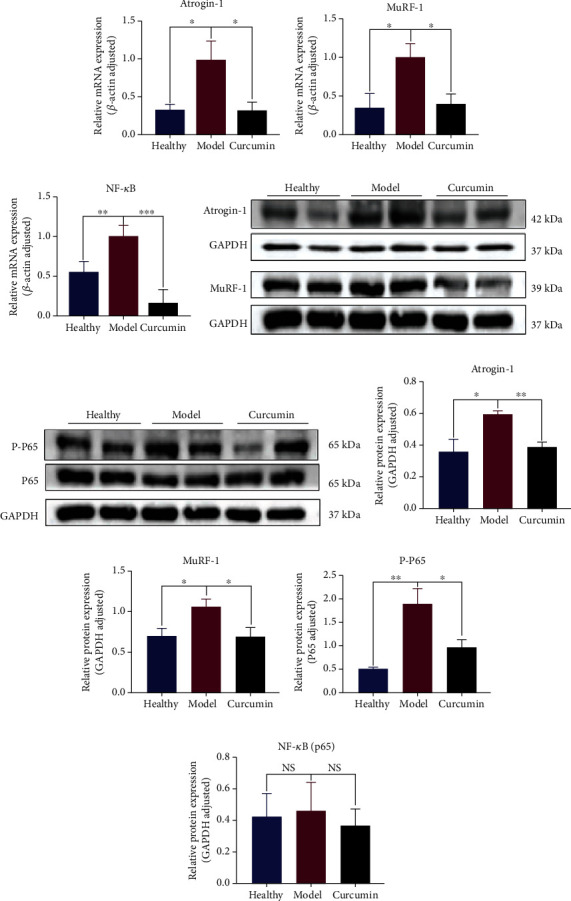
Curcumin treatment reduces ubiquitination and NF-*κ*B activity in muscle tissue of tumor-bearing mice. (a–c) Statistical analysis of atrogin-1, MuRF-1, and NF-*κ*B mRNA expression of mouse gastrocnemius in three groups. (d) Western blot bands of atrogin-1 and MuRF-1 protein in gastrocnemius of the three groups. (e) Western blot bands of P65 and P-P65 protein in gastrocnemius of the three groups. (f–i) Statistical analysis of atrogin-1, MuRF-1, P-65, and PP-65 protein expressions in gastrocnemius of three groups of mice. NS: not significant; ^∗^*p* < 0.05, ^∗∗^*p* < 0.01, and ^∗∗∗^*p* < 0.001 by *t*-test, compared to the model group. Data was shown as mean ± standard errors of the mean.

The ubiquitination-related gene expression was detected, and RT-qPCR results suggest that the expression of atrogin-1 and MuRF-1 in the model group was significantly higher than that in the healthy group (Figures 8(a) and 8(b); *p* = 0.0427, *p* = 0.0290), and this expression was significantly downregulated in the curcumin-treated group compared with the model group (Figures 8(a) and 8(b); *p* = 0.0098, *p* = 0.0378). The RT-qPCR analysis also observed that the NF-*κ*B in the gastrocnemius of the model group was significantly increased (Figure 8(c), *p* = 0.0028), while its expression in the curcumin group mice was downregulated compared with that in the model group (Figure 8(c), *p* = 0.0186). Western blot results for the detection of the protein expression of atrogin-1, MuRF-1, and p-NF-*κ*B p65 were consistent with the aforementioned RT-qPCR results (Figures 8(d)–8(i)).

## 4. Discussion

Most advanced cancer patients fit the category of tumor cachexia [27], and weight loss, especially the loss of skeletal muscle mass, is the main feature [28]. These features caused by tumor cachexia not only lead to poor tolerance of patients to multiple treatments but also have negative impact on the quality of life and prognosis of patients [29]. However, currently, there are very few methods or drugs to treat cancer cachexia. Curcumin is a food derivative with multiple biological targets and different cellular effects. It has anti-inflammatory, antioxidant, and anticancer properties. However, there are few studies on whether curcumin inhibits TNBC through these mechanisms and also reduces tumor cachexia-related skeletal muscle loss or weight loss. Given this, we conducted this study.

Dynamic bioluminescence imaging and tumor volume growth curve find that curcumin inhibited the growth and progression of TNBC tumor in tumor-bearing mice. These data strongly suggest that curcumin has an inhibitory effect on the growth of breast tumors and also suggest that curcumin has the potential to treat breast cancer cachexia. The gradual decrease in body weight is the earliest symptom of cachexia. In our study, 10 days after tumor inoculation in the model group, mouse weight and food intake began to be significantly lower than those of the healthy control group, and their hair color was dim, the spirit was listless, and vitality was decreased, which was consistent with the symptoms of cancer cachexia, while the weight and food intake of mice in the curcumin treatment group were gradually recovered from the 9th day of administration to the end of the experiment. The nutritional status is also a critically important factor of cancer cachexia [30]. Cholesterol and triglycerides are major sources of lipid energy in the body. Serum albumin serves as a nitrogen source to provide nutrition for tissues; serum total protein is often used to monitor the nutritional status of the body. The results showed that curcumin treatment significantly improved the malnutrition status; serum total protein, albumin, cholesterol, and triglyceride levels were higher than those in the model group. Therefore, it can be considered that curcumin not only inhibits the proliferation and progression of TNBC in tumor-bearing mice but also improves the nutritional status and weight loss, thus alleviating cancer cachexia.

Skeletal muscle loss is a typical feature of tumor cachexia [6]. The gastrocnemius in the calf muscles is the most common and earliest skeletal muscle involved, so we collected the gastrocnemius muscles of three groups of mice for downstream experiments. We found that the gastrocnemius muscle of the model group mice was significantly lost compared with that of the healthy group, but after curcumin treatment, the gastrocnemius muscle weight loss of the tumor-bearing mice was partially restored, and the gastrocnemius muscle index was higher than that of the model group mice. In addition, HE staining analysis of gastrocnemius muscle also found that the muscle fibers of the model group had smaller diameters and cross-sectional area, disordered arrangement, and significantly larger gaps, but these conditions were improved in the muscle fibers of the curcumin-treated mice. The forelimb grip and movement distance measurement of mice also confirmed the above results, the model group mice's movement distance per unit time was shortened, and the forelimb grip was continuously lost; these conditions were partially reversed after curcumin treatment. These results indicated the denser muscle fibers and stronger muscle strength of mice after curcumin administration.

The degradation of muscle protein caused by mitochondrial dysfunction is closely related to the skeletal muscle atrophy caused by cachexia [17, 31]. Uncoupling protein-2 and uncoupling protein-3 can destroy the inner and outer membrane of mitochondria, thereby reducing the mitochondrial ATP synthesis required for muscle energy metabolism [32]. In addition, the NF-*κ*B signaling pathway inhibits mitochondrial gene expression, thereby reducing mitochondrial biosynthesis, oxidative phosphorylation, and ATP production [33, 34]. We used TEM to observe the differences in mitochondrial morphology in the gastrocnemius muscles of different groups of mice, to verify whether curcumin reversed the loss of cachexia skeletal muscle is related to the improvement of mitochondrial function. The observation results suggested that there was significant mitochondrial dysfunction in atrophic muscle fibers of model mice compared with healthy mice, and curcumin intervention partially alleviated this phenomenon. Moreover, mitochondria are important productive organelles in cells, and mitochondrial dysfunction is bound to affect the production of ATP in muscles, leading to muscle atrophy [35]. In this study, except for the observed mitochondrial dysfunction in model mouse gastrocnemius muscles, the ATP content in the model mice was also decreased, but it was partly recovered in the curcumin group. These results indicated that the mitochondrial function of muscle fibers in mice treated with curcumin was improved, which also brought about an increase in ATP production.

Metabolic disorders are evident in the progression of skeletal muscle loss in patients with tumor dystrophy, so amelioration of specific metabolic defects is also a potential way to alleviate dystrophy [36]. We used ^1^HNMR metabonomic analysis to explore the changes of metabolites after curcumin improved muscle loss in dyscrasia mice, and a total of 6 significant differential metabolites were screened (AMP, lysine, isobutyrate,3-hydroxyphenylacetate, pyruvic acid, and creatine). AMP provides chemical energy by binding phosphate groups to form adenosine diphosphate (ADP) and adenosine triphosphate (ATP), and a reduction in AMP has been observed in atrophic muscle [37]. Lysine is a key substance that helps other nutrients to be fully absorbed and utilized by the human body. Carnitine deficiency caused by its lack is a common protein deficiency [38]. Isobutyrate is a kind of short-chain fatty acid. Studies have confirmed that the increase in butyrate synthesis by intestinal microbes is closely related to the increase in skeletal muscle index [39]. Fatty acids, glucose, and lactic acid can be metabolized into pyruvate, which is transported to the mitochondria by the mitochondrial pyruvate carrier (MPC) for the citric acid cycle [40]. Sufficient research has proved that creatine can promote muscle anabolism and is used as a supplement to reduce the loss of muscle mass and function in various acute and chronic diseases [41]. No studies have confirmed the effect of 3-hydroxyphenylacetate in alleviating muscle atrophy, and further studies are needed to confirm the effect. In KEGG analysis, these differential metabolites were enriched into the pyruvate metabolism, glycolysis/gluconeogenesis, and glyoxylate and dicarboxylate metabolism. Glycolysis/gluconeogenesis is also closely related to pyruvate metabolism. Pyruvate is an intermediate product of glucose metabolism, which is reduced to lactic acid for energy in the cytoplasm or oxidized into the mitochondria to generate acetyl-CoA and enter the TCA (tricarboxylic acid) cycle, realizing the mutual transformation of sugars, fats, and amino acids in the body [42]. Glyoxylic acid cycling improves the ability of organisms to utilize acetyl-CoA and is a reaction process closely related to the conversion of fats to carbohydrates. These three pathways are all related to the regulation of energy metabolism. Therefore, we believe that curcumin can reduce skeletal muscle loss through the regulation of energy metabolism in mice with cancer cachexia.

Inflammatory cytokines are thought to contribute to induce muscle atrophy and muscle metabolism disorders [43]. We found that the levels of IL-6 and TNF-*α* in gastrocnemius of mice in model group gastrocnemius were significantly higher than those in the healthy group, while curcumin partially inhibited elevated levels of IL-6 and TNF-*α* in tumor-bearing mice, which indicate the anti-inflammation ability of curcumin in TNBC-cachexia. Myostatin and myogenin are important regulators of muscle proteolysis and synthesis, and they play a key role in maintaining the steady-state balance of muscle mass [44]. Our results showed that myostatin expression was higher in the model group but dropped significantly in the curcumin group. Myogenin had opposite results. Therefore, we suggest that curcumin may reduce the expression of muscle degrading factor myostatin and increase the myogenic factor myogenin to achieve the effect of reducing cachexia-related muscle atrophy.

Degradation of muscle proteins through the ubiquitin-proteasome pathway is also a form of muscle atrophy. Previous studies have shown that atrogin-1 and MuRF-1, two muscle-specific E3 ubiquitin ligases, are overexpressed in skeletal muscle in cancer-induced muscle atrophy [45, 46]. In addition, *in vitro* experiments have shown that increased myostatin is often accompanied by increased MAFbx expression [47]. We used RT-qPCR and WB analysis to verify whether curcumin alleviated muscle loss in mice with tumor cachexia by reducing the level of ubiquitination. The results showed that the expression levels of atrogin-1 and MuRF-1 in the gastrocnemius muscle of the model group were significantly higher than those in the healthy group; curcumin reversed this increasing trend. It was consistent with these previous reports and also indicated a regulation role of curcumin on atrogin-1 and MuRF-1. The release of inflammatory factors in the tumor microenvironment can activate the NF-*κ*B pathway, which not only directly induces muscle atrophy but also mediates the transcription of atrogin-1 and MuRF-1, leading to increased protein degradation, which indicates that NF-KB is expected to become a therapeutic target for muscular dystrophy [20, 48]. The RT-qPCR analysis found that NF-*κ*B transcription expression was increased in the model group, while the curcumin group decreased its expression. Western blot results of phosphorylated NF-*κ*B (P-P65) observed the similar tendency. Therefore, we speculate that activation of the NF-*κ*B pathway is one of the mechanisms of curcumin in reducing tumor cachexia and muscle loss.

**Figure 9:**
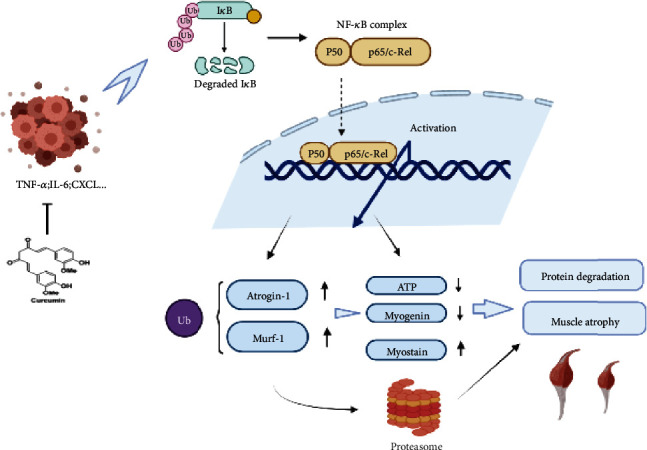
Therapeutic mechanism of curcumin on muscle atrophy in triple-negative breast cancer cachexia.

Based on the above results, we demonstrate that curcumin not only inhibits the proliferation of TNBC but also reduces the weight and skeletal muscle loss associated with tumor-cachexia. The experimental conclusions suggest that its anticachexia effect may be through reducing the expression of inflammatory factors, inhibiting the activation of the NF-*κ*B pathway, reducing the level of ubiquitination in muscle tissue, alleviating fiber mitochondrial dysfunction, improving the malnutrition status and the energy metabolism of muscle tissue reduces the degradation of muscle and increases its synthesis (Figure 9). These mechanisms interact with each other and work together to improve the quality and function of skeletal muscle.

## 5. Conclusion

In conclusion, curcumin not only inhibits the proliferation of TNBC but also targets the NF-*κ*B/UPS axis ameliorating muscle atrophy in TNBC-cachexia mice.

## Figures and Tables

**Table 1:** Primers' table.

Gene name	Forward primer	Reverse primer
*β*-Actin	TGCTGTCCCTGTATGCCTCTG	TGATGTCACGCACGATTTCC
Myostatin	AGTGGATCTAAATGAGGGCAGT	GTTTCCAGGCGCAGCTTAC
Myogenin	GAGACATCCCCCTATTTCTACCA	GCTCAGTCCGCTCATAGCC
Atrogin-1	CAGCTTCGTGAGCGACCTC	GGCAGTCGAGAAGTCCAGTC
MuRF1	GTGTGAGGTGCCTACTTGCTC	GCTCAGTCTTCTGTCCTTGGA
Nk-kB	AGCGGGAACTGAGTGAGATGA	GCACCCAGGTTGTATCGGG

## Data Availability

The datasets used and/or analyzed during the current study are available from the corresponding author on reasonable request.

## Abbreviations

ADP: Adenosine diphosphate
AMP: Adenosine monophosphate
ATP: Adenosine triphosphate
BCA: Bicinchoninic acid
ELISA: Enzyme-linked immunosorbent assay
FDR: False discovery rate
FIDs: Free induction decays
gf: Gram force
HE: Hematoxylin-eosin
^1^HNMR: H-nuclear magnetic resonance
IL-6: Interleukin-6
KEGG: Kyoto Encyclopedia of Genes and Genomes
MPC: Mitochondrial pyruvate carrier
NF-*κ*B: Nuclear factor-k-gene binding
PLS-DA: The partial least-squares discriminant analysis
PVDF: Polyvinylidene fluoride
RT-PCR: Reverse transcription-polymerase chain reaction
SDS-PAGE: Sodium dodecyl sulfate-polyacrylamide gel electrophoresis
SEM: Standard error of the mean
TCA cycle: Tricarboxylic acid cycle
TEM: Transmission electron microscopy
TNBC: Triple-negative breast cancer
TNF-*α*: Tumor necrosis factor *α*
UPS: Ubiquitin-proteasome system
VIP: Variable influence on projection
WB: Western blot.
