# Supplementary material for: Curcumin Targeting NF-κB/Ubiquitin-Proteasome-System Axis Ameliorates Muscle Atrophy in Triple-Negative Breast Cancer Cachexia Mice
Source: Mediators Inflamm. 2022 Jan 29;2022:2567150. doi: 10.1155/2022/2567150 (PMC8817892; doi:10.1155/2022/2567150)
Supplement: Supplementary Materials — Supplementary Table S1 is provided. [file 2567150.f1.docx]

Supplementary tables S1

|  | total | expected | hits | Raw p | Holm p | FDR |
| --- | --- | --- | --- | --- | --- | --- |
| Pyruvate metabolism | 22 | 0.13 | 2 | 0.00676 | 0.567 | 0.313 |
| Glycolysis / Gluconeogenesis | 26 | 0.154 | 2 | 0.00939 | 0.779 | 0.313 |
| Glyoxylate and dicarboxylate metabolism | 32 | 0.189 | 2 | 0.0141 | 1 | 0.313 |
| Glycine, serine and threonine metabolism | 33 | 0.195 | 2 | 0.0149 | 1 | 0.313 |
| Arginine and proline metabolism | 38 | 0.225 | 2 | 0.0196 | 1 | 0.329 |
| Biotin metabolism | 10 | 0.0591 | 1 | 0.0578 | 1 | 0.809 |
| Citrate cycle (TCA cycle) | 20 | 0.118 | 1 | 0.113 | 1 | 1 |
| Lysine degradation | 25 | 0.148 | 1 | 0.139 | 1 | 1 |
| Alanine, aspartate and glutamate metabolism | 28 | 0.166 | 1 | 0.154 | 1 | 1 |
| Cysteine and methionine metabolism | 33 | 0.195 | 1 | 0.179 | 1 | 1 |
| Tyrosine metabolism | 42 | 0.248 | 1 | 0.223 | 1 | 1 |
| Aminoacyl-tRNA biosynthesis | 48 | 0.284 | 1 | 0.251 | 1 | 1 |
| Purine metabolism | 65 | 0.384 | 1 | 0.326 | 1 | 1 |
